# Association between body condition score and abdominal fat assessed by ultrasound in Jersey cows

**DOI:** 10.1002/vms3.1515

**Published:** 2024-08-29

**Authors:** Pedro Melendez, Daniela Redrovan, Prasanth K. Chelikani

**Affiliations:** ^1^ Jockey Club College of Veterinary Medicine & Life Sciences City University of Hong Kong Hong Kong SAR China; ^2^ School of Veterinary Medicine Texas Tech University Amarillo Texas USA

**Keywords:** abdominal fat, body condition score, cattle, Jersey, ultrasound

## Abstract

**Background:**

Body condition score (BCS) is a subjective tool and the deposition of subcutaneous fat differs from the deposition of abdominal fat.

**Objective:**

The aim of this study was to evaluate by multivariate regression models and ultrasonography the amount of fat accumulated in different areas of the abdominal cavity in Jersey non‐pregnant non‐lactating cows and its association with their BCS.

**Methods:**

From a commercial farm, 21 non‐pregnant non‐lactating Jersey cows were selected at random. Cows were placed in a headlock line, and BCS was evaluated (scale 1–5 with a 0.25 unit increment) by the same assessor. Ultrasonographic evaluation was performed using a Sonosite machine in duplicate, considering key anatomical points of the body to predict total abdominal fat (AT), retroperitoneal fat (RT), omental fat (OT) and mesenteric fat (MT). A regression analysis for each abdominal fat depot and the BCS was run using SAS.

**Results:**

Models from the lowest to the largest *r* ^2^ are reported. The *r* ^2^ for the models were MT *r* ^2^ = 0.023; RT *r* ^2^ = 0.1047; OT *r* ^2^ = 0.323 and AT *r* ^2^ = 0.369. Correlation between BCS and abdominal fat depots was positive, lower for mesenteric and retroperitoneal fat, but higher for omental and total abdominal fat. Cows were divided on the basis of the median of BCS distribution to high (≥3.5) and low (≤3.25). Those with high BCS had significantly larger amounts of fat in different anatomical areas of abdominal cavity than cows with low BCS.

**Conclusion:**

BCS has a low‐to‐moderate association with abdominal fat depots, but a high variability exists within each BCS punctuation, which supports the notion that fat accumulation patterns and metabolic turnover between abdominal and subcutaneous fat differ from each other.

## Graphical abstract

Abdominal fat deposits (retroperitoneal, omental and mesenteric) in non‐lactating non‐pregnant Jersey cows were estimated by regression mixed models using ultrasonography assessment of external anatomical areas (subcutaneous fat), and retroperitoneal layers were moderately associated with body condition score. These findings suggest that cows with fair body condition scores (3.25–3.5) still can have large amounts of abdominal fat deposits, implying that they can be at higher risk of developing metabolic diseases such as fatty liver, ketosis and displacement of the abomasum.

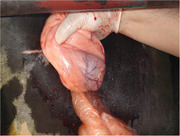

## 1 INTRODUCTION

The transition period in dairy cows is defined as the time from 3 weeks prepartum to 3 weeks post‐partum, which is characterized by considerable metabolic and endocrine adjustments that cows must experience from the end of gestation to the beginning of lactation (Drackley et al., 2001; Melendez & Risco, 2021).

One of the characteristics of the transition period is the reduction in dry matter intake (DMI) as calving approaches. Furthermore, the beginning of lactation increases energy requirements to meet the demands of milk production. As a result, the cow experiences a typical negative energy balance (NEB) during the peripartum period characterized by the activation of lipases that release non‐esterified fatty acids (NEFA) into the bloodstream from triglycerides in adipose tissue (Contreras et al., 2015, 2018; De Koster & Opsomer, 2013; Drackley et al., 2001). Therefore, the main function of adipose tissue is to store and release lipids in response to energy demands. However, adipose tissue also has immune, endocrine, regenerative, mechanical and thermal functions, which differ depending on the types of fat depots (Contreras et al., 2015, 2018; Tchkonia et al., 2013). In fact, subcutaneous fat has more unsaturated fatty acids than abdominal fat (De Smet et al., 2004). On the other hand, visceral adipocytes are more metabolically active and sensitive to lipolysis than subcutaneous adipocytes due to greater gene expression of the hormone‐sensitive lipase (Contreras et al., 2018; Locher et al., 2011). Therefore, cows that deposit more fat in the abdominal cavity than in the subcutaneous tissue have a greater risk of releasing a larger amount of NEFA and presenting an increase in diseases such as fatty liver, displaced abomasum (DA) and/or ketosis (Contreras et al., 2018).

The distribution of body fat varies depending on gender, genetics, diseases, hormones and ageing (Tchkonia et al., 2013). In fact, in a study carried out in the USA, it was reported that cows with an excessive amount of omental and total abdominal fat had an important genetic component. It was concluded that excess of omental/abdominal fat in Holstein cows with similar body condition score (BCS) is at least moderately heritable (Melendez et al., 2019). In another study conducted in the USA, a genomic prediction for DA was also found in Holstein cows (McNeel et al., 2017). In a more recent study that analysed 28,000 disease records from 14,000 lactations, it was found that a region on bovine chromosome 20 was significantly associated with the accumulation of visceral fat as well as with the development of DA. Overall, it was concluded that visceral fat deposition in dairy cows is controlled by both additive and non‐additive effects and that at least one genomic region had pleiotropic effects on both visceral fat deposition and DA development (Novo et al., 2022). With this evidence, it is suggested that visceral adiposity is a key factor in the presentation of metabolic diseases and inflammatory processes in the modern dairy cow (Contreras et al., 2018; Ji et al., 2014; Novo et al., 2022).

Having clear evidence that abdominal adiposity is also related to milk production, as it is a more active fat to provide quick energy to the cow, it is imperative to understand the physiology and metabolism of abdominal fat in more detail. Because selection for milk yield and solid production has brought genes that are associated with greater fat depots in the abdominal cavity, it is not easy to carry out genetic selection against abdominal adiposity as it could affect genetic improvement for milk production. Consequently, a better knowledge of the physiology and metabolic dynamics of abdominal fat and the evolution of certain diseases will provide the basis for data to establish better control and prevention of these metabolic conditions (Melendez & Risco, 2021; McNeel et al., 2017; Novo et al., 2022). On the other hand, as the evaluation of BCS is a subjective tool and the deposition of subcutaneous fat differs from the deposition of abdominal fat, the objective of this study was to estimate the amount of fat accumulated in different areas of the abdominal cavity using ultrasonography in non‐lactating non‐pregnant Jersey cows with different BCSs. The hypothesis of this study was that cows with the same BCS have a significant variation in their abdominal fat content, reflecting that at the same BCS, some cows have more abdominal fat and therefore may have a greater risk of developing metabolic diseases such as fatty liver, ketosis and DA.

## 2 MATERIALS AND METHODS

### 2.1 Animals

From a commercial farm in the USA, 21 non‐pregnant, non‐lactating, 5–8‐year‐old Jersey cows were randomly selected. The cows were transported to an experimental unit. The cows, upon arrival, had a BCS of 2.75–3.75. They were fed daily with alfalfa hay and water at discretion.

### 2.2 Experimental design

In December 2020, cows were placed on a headlock system line to be evaluated for BCS using a scale of 1–5 with a 0.25 unit increment (Angeli et al., 2021; Melendez et al., 2020). The assessment of BCS was carried out by the same evaluator who, in turn, performed an ultrasound assessment to predict the accumulation and deposition of abdominal fat, using a Sonosite equipment (SonoSite M‐Turbo). Ultrasound was performed two times on consecutive days, and the results were averaged. Ultrasound was performed at key anatomical points of the body to predict total abdominal fat (AT), retroperitoneal fat (RPAT), omental fat (OMAT) and mesenteric fat (MAT) using the methodology of Raschka et al. (2016). The authors of this research concluded that ultrasonographic measurement of the subcutaneous and retroperitoneal fat layers seems sufficiently precise for the clinical evaluation of the amounts of subcutaneous adipose tissue, AT, RPAT, OMAT and MAT in cows. Seven ultrasound measurements at six anatomical locations are required to determine the different areas of abdominal fat deposition. This technique facilitates the serial evaluation of the distribution and dynamics of adipose tissue over time during the production cycle of dairy cows. This method could be used to investigate the pathogenic role of visceral adipose tissue depots in dairy cows.

Seven anatomical areas were measured to determine the amount of adipose tissue:

1. R12 is the subcutaneous fat over the 12th rib.
2. BFT is the back fat thickness.
3. AW1b is the distance from the skin to the distal muscular margin above the peritoneum at the point of intersection of a vertical line passing through the last lumbar vertebra and a horizontal line passing through the patella.
4. AW3b is the distance from the skin to the distal muscular margin above the peritoneum in the centre of the paralumbar fossa.
5. AW3c is the thickness of the abdominal wall in the centre of the paralumbar fossa.
6. KD2c is the distance from the skin to the peritoneum in the intertransverse space directly cranial to the intertransverse space where the caudal pole of the kidney is visible.
7. KD3b is the distance from the skin to the distal margin of the kidney in the intertransverse space directly cranial to KD2.

Table 1 summarizes the multivariable models according to Rascha et al. (2016) used to estimate the amount of fat in the different abdominal fat depots.

**TABLE 1:** Multivariable models according to Raschka et al. (2016) used to estimate the amount of fat in the different abdominal deposits.

Parameter estimate		AT			RPAT			OMAT			MAT	
*p‐Value*		<0.001			<0.001			<0.001			<0.001	
*r* ^2^		0.94			0.94			0.84			0.94	

Variable	Parameter	SE	*p*‐Value	Parameter	SE	*p*‐Value	Parameter	SE	*p*‐Value	Parameter	SE	*p*‐Value
Intercept	−39.5	5.65	<0.001	−9.55	1.61	<0.001	−2.32	1.70	0.19	−12.8	1.92	<0.001
R12	1.02	0.26	0.001	0.62	0.05	<0.001						
BFT							0.55	0.20	0.013			
AW1b	0.92	0.22	<0.001							0.38	0.09	<0.001
AW3b							0.37	0.09	<0.001	1.73	0.64	0.015
AW3c										−1.45	0.62	0.031
KD2c	0.25	0.05	<0.001							0.07	0.02	<0.001
KD3b				0.06	0.01	<0.001						

*Note*: As an example, for total abdominal fat (AT) the model to use was: Intercept + R12(*X*1) + AW1b(*X*2) + KD2c(*X*3) which is equivalent to −39.5 + 1.02(*X*1) + 0.92(*X*2) + 0.25(*X*3), where *X*1, *X*2 and *X*3 are the values of the ultrasonographic measurements of the three respective anatomical areas in millimetres to predict the total abdominal fat in kilograms.

Abbreviations: AT, total abdominal fat; MAT, mesenteric fat; OMAT, omental fat; RPAT, retroperitoneal fat.

Table 2 presents the results of ultrasonography of the anatomical areas recommended to estimate the amount of adipose tissue of the different abdominal fat depots in the cows under study.

**TABLE 2:** Results of ultrasonographic measurements in duplicate and averaged in millimetres and body condition score (BCS) of the 21 non‐pregnant non‐lactating cows.

Cow	BCS	AW1b	R12	Kd2c	KD3b	BF	AW3b	AW3c
1	3.5	28.8	6.2	131	120	6.2	15.7	18
2	3.25	15.3	3.1	94.1	83.8	5	9.3	12.8
3	3.5	18	10.5	122	88.5	12.2	16.2	19.1
4	3.5	19.1	20.5	119	115	12.6	12.2	15.6
5	3.5	20.4	19.3	90.6	80.8	9.5	12.6	16
6	3.5	28.3	15.5	121	109	11.8	16.2	21.1
7	3.5	28.1	10.6	102	101	7.9	23	30.5
8	3.75	19.3	21.1	101	91.4	12.9	21.1	26.2
9	2.75	15.1	13.5	83.3	80.7	5.7	11.1	15.1
10	3	12	10.5	128	122	8.7	9.6	11.6
11	3.25	24.4	10	74.2	73.2	8	20	22
12	3.5	19.8	8.4	119	114	11.8	15.8	18.9
13	2.75	15.5	13.6	107	97.2	9.1	12.2	15.5
14	3.5	10.7	16.2	147	132	12.5	24.2	26.4
15	2.75	7.9	6.4	108	109	9.3	13.1	15.1
16	3.5	14.7	8	101	88.5	13.3	18.4	20.2
17	3.25	13.1	6.4	122	115	9.3	19.4	22.2
18	3.75	14.5	8.5	141	135	12.2	29.3	32.7
19	3	16.5	6.7	105	105	8.4	28	30.9
20	3.5	19.1	11.6	124	120	9.8	15.6	17.6
21	3.75	16.9	9.1	132	118	11.3	17.9	20

Photo 1 shows as an example the ultrasound evaluation of subcutaneous fat at the level of the 12th rib (R12), which is a parameter estimate of the multivariable model for the AT fat based on Raschka et al. (2016).

### 2.3 Statistical analysis

Once the amount of fat was obtained according to the adipose tissue deposition models (ATT, RPAT, OMAT and MAT), a multivariable regression analysis was performed to establish the association of each abdominal fat depot with BCS, using the SAS statistical package 9.4 (SAS). Multivariable regression models are reported according to their *r* ^2^. Two groups of cows were created using the median of the BCS distribution. Cows with low BCS (≤3.25) and high BCS (>3.25) were compared in terms of the amount of abdominal fat deposition predicted by the statistical models. Each cow was considered a random variable nested in its group. For all models, the best fit was specified according to the value of the Schwarz Bayesian Criterion. LSMEANS were calculated and reported (Littell et al., 1998, 1996).

## 3 RESULTS

According to the different models, the relationship between BCS and abdominal fat depots showed an *r* ^2^ that was lower for MAT and RPAT (2.3% and 13%, respectively), but higher for OMAT and AT (36% and 37%, respectively).

### 3.1 BCS and retroperitoneal fat

**FIGURE 1:**
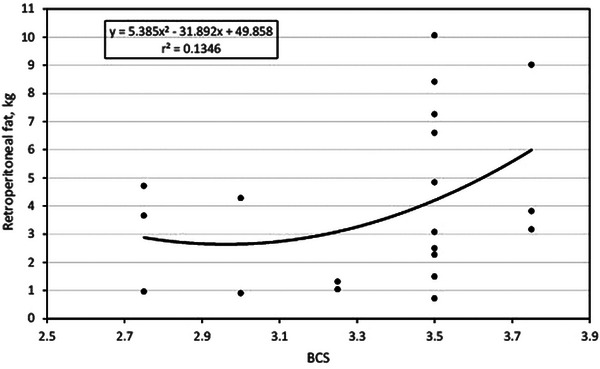
Relationship between body condition score and retroperitoneal fat.

Figure 1 plots the association between BCS and RPAT fat in the study cows. The association was a second‐order polynomial curvilinear type where RPAT fat was equal to 5.385(*X*)^2^ − 31.892(*X*) + 49.858. As an example, it is observed that in cows with BCS 3.5, the RPAT fat was from less than 1 kg to more than 10 kg, showing a large variability in cows with the same BCS. Furthermore, according to the trend curve, it is observed that RPAT fat practically varies between 1 and 5 kg in cows with BCS from 2.75 to 3.25, and then the variation increases at BCS 3.5. This association is moderate as the *r* ^2^ was 0.13.

### 3.2 BCS and mesenteric fat

**FIGURE 2:**
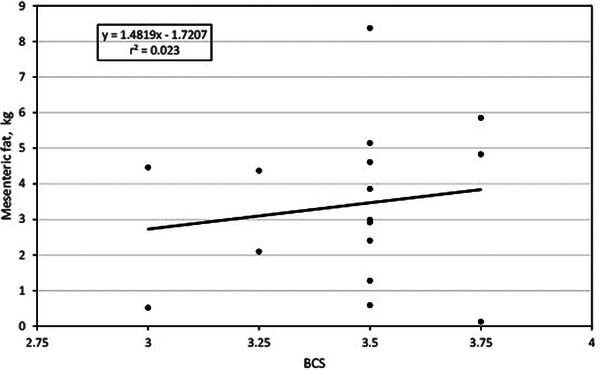
Relationship between body condition score and mesenteric fat.

Figure 2 shows the association between BCS and MAT in the study cows. The association was linear where retroperitoneal fat was equal to 1.4819(*X*) − 1.7207. Although the association is minor as the *r* ^2^ is 0.023, it is seen that MAT fat varies considerably in cows within the same BCS, being much more evident in cows with BCS of 3.5 where the MAT fat was estimated to a range from less than 1 kg to more than 8 kg.

### 3.3 BCS and omental fat

**FIGURE 3:**
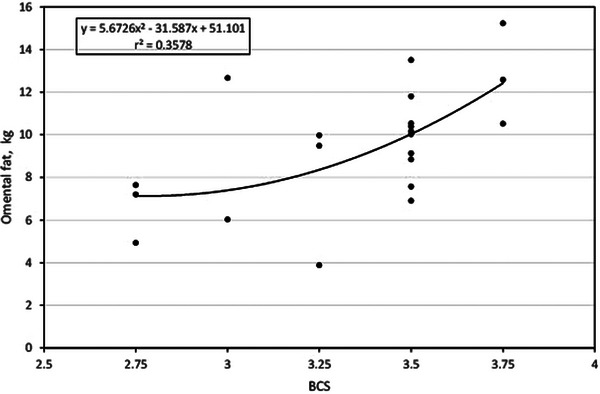
Relationship between body condition score and omental fat.

Figure 3 shows the association between BCS and OMAT fat in the study cows. The association was a second‐order polynomial curvilinear type of moderate‐to‐medium strength with an *r* ^2^ = 0.36, where OMAT fat was equal to 5.6726(*X*)^2^ − 31.587(*X*) + 51.101. According to the trend curve, OMAT fat varies between 5 and 8 kg in cows with BCS 2.75, and then the variation increases at BCS 3.25 with OMAT fat between 4 and 10 kg and in cows with BCS 3.5 with OMAT fat between 7 and 14 kg.

### 3.4 BCS and total abdominal fat

**FIGURE 4:**
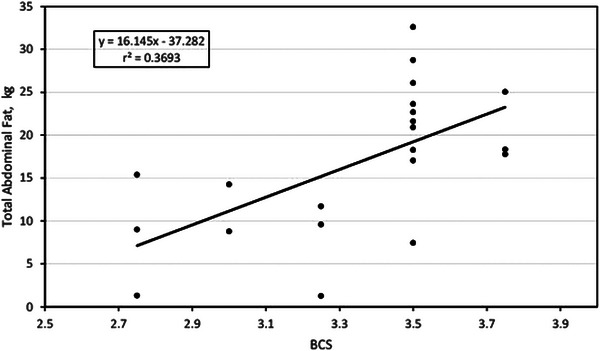
Relationship between body condition score and total abdominal fat.

Figure 4, 5 plots the association between BCS and AT fat in the study cows. The association was linear where AT fat was equal to 16.145(*X*) − 37.282. The association is moderate to strong with an *r* ^2^ of 0.37. It is very evident that AT fat also varies consistently in cows with the same BCS, being much more manifest in cows with BCS 3.5 where AT fat was estimated between less than 7 kg and more than 32 kg.

**FIGURE 5:**
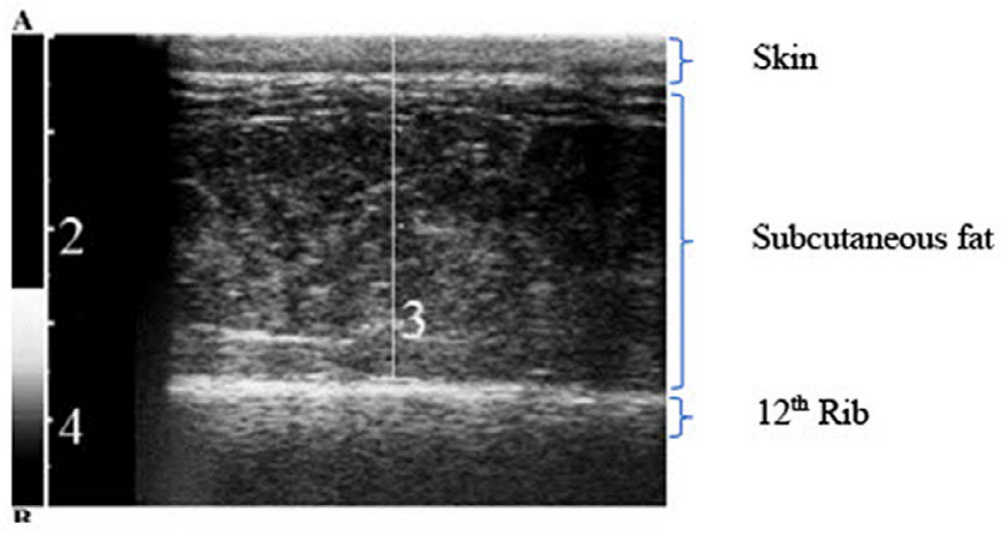
Ultasonographic measurement of subcutaneous fat at the level of the 12th rib to obtain one of the parameters of the multivariable models to estimate total abdominal fat and retroperitoneal fat (Raschka et al., 2016).

### 3.5 Abdominal fat in cows with high and low BCS

Cows were divided according to the median value of the BCS distribution into high (≥3.5) and low (≤3.25) BCS. Cows with high BCS had significantly greater amounts of fat deposited in the different anatomical areas of the abdominal cavity than cows with low BCS. Table 3 shows the amount of AT, RPAT, OMAT and MAT fat in the study cows with BCS ≤3.25 (*n* = 8) and BCS ≥3.50 (*n* = 13). It is observed that the differences in the content of all fat depots were significant (*p* ≤ 0.05) with greater amounts of fat in cows with higher BCS than in cows with lower BCS.

**TABLE 3:** Total abdominal, retroperitoneal, omental and mesenteric fat (mean ± SEM) in non‐lactating, non‐pregnant Jersey cows with BCS ≤3.25 and BCS ≥3.50.

BCS (1–5)	AT (kg)	RPAT (kg)	OMAT (kg)	MAT (kg)
≤3.25 (*n* = 8)	8.90 ± 1.86	2.41 ± 0.63	7.72 ± 0.62	1.86 ± 0.48
≥3.50 (*n* = 13)	21.5 ± 1.96	4.86 ± 0.72	10.6 ± 0.70	3.57 ± 0.49
*p‐Value*	<0.001	0.03	0.01	0.04

Abbreviation: BCS, body condition score.

## 4 DISCUSSION

This study aimed to determine the amounts of fat deposited in different areas of the abdominal cavity using indirectly ultrasonographic evaluation of certain anatomical areas of the body and predictive equations and models that contemplate the information obtained by ultrasound. Cows with different BCSs were considered to have variability that allows differences to be obtained. However, one of the hypotheses of the following investigation was that the accumulation of fat in certain areas of the abdominal cavity varies consistently even in animals with the same BCS. Without a doubt, this aspect was the most important feature in the development of this research, as cows with the same BCS presented a high variability in the accumulation of abdominal fat, which may help explain why even animals with adequate BCS at calving can still develop metabolic diseases as a result of this excess abdominal fat. This fat is metabolically much more active and pro‐inflammatory than subcutaneous fat (Contreras et al., 2018; Tchkonia et al., 2013). In addition, these metabolic disorders also represent important economic losses for the dairy industry, which are characterized by a more severe rate of lipolysis and an accumulation of macrophages that present a phenotype in a more pro‐inflammatory state (macrophage M1) than an inflammatory cell in resolution (macrophage M2). Furthermore, elevated levels of NEFA have been associated with evident immunosuppression by affecting the normal activity of neutrophils and the inflammatory response of macrophages and lymphocytes (Contreras et al., 2018; Häussler et al., 2013; Locher et al., 2011). On the other hand, ruminants with severe post‐partum lipolysis have increased expression of type I and III collagens in adipose tissue. Concomitantly, the expression of thrombospondin 1, a protein that plays an important role in the extracellular matrix of cells, is also upregulated during the early post‐partum period, when high rates of lipolysis occur. Both collagen type III and thrombospondin 1 expressed in high amounts in adipose tissue have been linked to insulin resistance in fat cells, which may further exacerbate the development of fatty liver (Contreras et al., 2018; De Koster & Opsomer, 2013; De Smet et al., 2004; Locher et al., 2011; Tchkonia et al., 2013). With this evidence, it is suggested that visceral adiposity is a key factor in the presentation of metabolic diseases and inflammatory processes in the modern dairy cow (Contreras et al., 2018; Ji et al., 2014; Novo et al., 2022). Furthermore, the evaluation of BCS is a subjective tool that is also not able to properly detect the variability in abdominal fat deposition. This comes to reconsider the use of BCS evaluation as the only method to monitor energy nutrition on dairy cattle. Within this, it is imperative to consider other types of tools and biological markers that allow us to examine animals that, even with adequate BCS, may be mobilizing body fat more severely and developing ketosis and DA (Häussler et al., 2013; Hostens et al., 2013; Ioannidis & Donadeu, 2018; Melendez et al., 2017, 2018; Mömke et al., 2013; Shen et al., 2018).

Overall, the results obtained in this research are consistent because it was expected that cows with higher BCS would have a greater content of abdominal adipose tissue (Table 3); however, this relationship is not very strong as the multivariable regression models varied from a small *r* ^2^ (0.023) to a moderate *r* ^2^ (0.37). This means that less than 50% of the variation in abdominal fat is explained by the variation in BCS. Furthermore, when analysing the regression trends, it is manifest the great variability in abdominal fat that the animals present within the same BCS. In the case of RPAT fat, in cows with BCS 3.5, a variation of 1000% is observed, as there are cows with less than 1 kg and cows with 10 kg of RPAT fat. The same goes for the rest of the types of abdominal depots.

However, one of the weaknesses of this study is the use of predictive mathematical models obtained from other research carried out under different condition research settings. The ideal would have been to have a more precise prediction of abdominal fat content, perhaps considering more radical methods such as evaluating postmortem animals by directly determining the total amount of all fat depots. This alternative was not feasible due to financial limitations of the study and ethical restrictions imposed by the University Animal Use Committee for the slaughtering of animals according to the current experimental design. However, the use of predictive mathematical models has this advantage by considering measurable variables in vivo, such as ultrasonography and prediction of a secondary variable such as intra‐abdominal fat deposition. Perhaps a secondary measurement such as the assessment of blood metabolites for the determination of NEFA, BHB, fatty acid profile, pro‐inflammatory cytokines, to name a few, would have been a complementary tool to more accurately evaluate the physiology of the adipose tissue of the cows under study. However, the cows were not pregnant and not producing milk (dry cows) with a maintenance diet; therefore, the cows were most likely not on an NEB; therefore, fat mobilization was unlikely. Despite these interpretations and shortcomings, our study only had the objective of indirectly estimating the intra‐abdominal fat content in cows with different BCS and within animals with the same BCS under not productive stress. Notwithstanding, the shortcomings of the current investigation are points to be taken into consideration for future research that allows improving experimental design and more precise estimation of the variables to be studied.

It is concluded that BCS has a low‐to‐moderate association with abdominal fat depots, which supports the idea that patterns of fat accumulation and metabolic turnover between abdominal and subcutaneous fat differ from each other. Animals with higher BCS had a greater intra‐abdominal fat content than animals with low BCS; however, animals with the same BCS also had a consistent variation in their intra‐abdominal fat depots.

## AUTHOR CONTRIBUTIONS

Pedro Melendez contributed to the study design, ultrasonography, record keeping, statistical analysis and manuscript writing and editing. Prasanth K. Chelikani contributed to the study design, ultrasonography, record keeping and manuscript writing and editing. Daniela Redrovan contributed to the ultrasonography, record keeping and manuscript writing and editing.

## CONFLICT OF INTEREST STATEMENT

The authors declare no conflicts of interest.

## FUNDING INFORMATION

School of Veterinary Medicine of the Texas Tech University.

### PEER REVIEW

The peer review history for this paper is available at https://publons.com/publon/10.1002/vms3.1515.

## ETHICS STATEMENT

The study was conducted under the standards of the Animal Care & Use Research Committee of the Texas Tech University.

## INFORMED CONSENT

Informed consent was not required. The animals belonged to the Texas Tech University.

## Data Availability

All data generated or analysed during this study are available upon request.
